# Synthesis and Characterization of a Novel Green Cationic Polyfluorene and Its Potential Use as a Fluorescent Membrane Probe

**DOI:** 10.3390/polym10090938

**Published:** 2018-08-23

**Authors:** Rebeca Vázquez-Guilló, María José Martínez-Tomé, Zehra Kahveci, Ivan Torres, Alberto Falco, Ricardo Mallavia, C. Reyes Mateo

**Affiliations:** 1 Instituto de Biología Molecular y Celular, Universidad Miguel Hernández, 03202 Elche, Spain; rvazquez@umh.es (R.V.-G.); mj.martinez@umh.es (M.J.M.-T.); alber.falco@umh.es (A.F.); 2 Living Systems Institute, University of Exeter, Exeter EX4 4QD, UK; z.kahveci@exeter.ac.uk; 3 Departamento de Química Inorgánca, Orgánica y Bioqímica, Universidad de Castilla la Mancha, 13071 Cuidad Real, Spain; Ivan.TorresMoya@uclm.es

**Keywords:** polyfluorene, conjugated polyelectrolyte, bioimaging, fluorescent membrane probe, bioimaging

## Abstract

In the present work, we have synthesized a novel green-emitter conjugated polyelectrolyte Copoly-{[9,9-bis(6′-*N*,*N*,*N*-trimethylammonium)hexyl]-2,7-(fluorene)-*alt*-4,7-(2-(phenyl) benzo[d] [1,2,3] triazole)} bromide (HTMA-PFBT) by microwave-assisted Suzuki coupling reaction. Its fluorescent properties have been studied in aqueous media and in presence of model membranes of different composition, in order to explore its ability to be used as a green fluorescent membrane probe. The polyelectrolyte was bound with high affinity to the membrane surface, where it exhibited high fluorescence efficiency and stability. HTMA-PFBT showed lower affinity to zwitterionic membranes as compared to anionic ones, as well as a more external location, near the membrane-aqueous interface. Fluorescence microscopy studies confirmed the interaction of HTMA-PFBT with the model membranes, labelling the lipid bilayer without perturbing its morphology and showing a clear preference towards anionic systems. In addition, the polyelectrolyte was able to label the membrane of bacteria and living mammalian cells, separately. Finally, we explored if the polyelectrolyte can function also as a sensitive probe able of detecting lipid-phase transitions. All these results suggest the potential use of HTMA-PFBT as a green membrane marker for bioimaging and selective recognition of bacteria cell over mammalian ones and as a tool to monitor changes in physical state of lipid membranes.

## 1. Introduction

Over the last several decades fluorescent probes have become essential tools in biology and biotechnology research allowing the labelling and imaging of non-fluorescent biological systems as well as providing information on specific molecular processes [1,2]. The selection of the probe depends on the type of sample to be explored, the optical technique and the question asked [3,4]. Generally, probes should have high extinction coefficient and fluorescent quantum yield, showing high affinity to the biological target of interest, without perturbing it. In addition, they should have high water solubility, photostability and be easy for bioconjugation, as well as have good biocompatibility and be easily adaptable to different experimental conditions and should not be affected by changes in ionic strength, pH or temperature. Most of fluorescent probes currently used are based on either small organic dyes or luminescent proteins. However, these fluorescent materials show some important restrictions like photobleaching, self-quenching and chemical decomposition, which limit their applications [5,6]. Quantum dots (QDs) offer a logical alternative due to their interesting properties. They are powerful and versatile fluorescent nanoparticles able to label and penetrate into cells without losing their unique photophysical properties (tunable emission from blue to near-infrared regions, high fluorescence quantum yield and photostability) [7]. However, there is a major concern for potential cytotoxicity risk of QDs associated with their heavy metal components [8].

More recently, fluorescent probes based on nanoparticles other than QDs and conjugated polymers (CPs) have been introduced [1,9,10,11,12,13]. CPs seem to be interesting candidates for designing alternative fluorescent probes due to their unique properties. These are carbon based macromolecules composed of repeating monomeric units forming a chain, the backbone, which contains an alternating sequence of single and double bonds, a π-conjugated system, where electrons are effectively delocalized over the length of the chain. It is precisely this alternating bond sequence that enables CPs to exhibit their unique optical and electronic properties [14,15].

The synthesis of CPs by coupling reactions usually requires long reaction times. In recent years, usage of microwaves in organic synthesis has increased exponentially, mainly because it drastically reduces the reaction time, improves reproducibility and provides greater control over reaction conditions. Thus, nowadays microwave synthesis is widely used for the organic synthesis of numerous compounds, including polymers [16,17]. To date, few examples of CPs prepared in microwave have been published [18,19,20,21]. Scherf and colleagues published the first reaction of these polymers by cross-coupling Suzuki in microwave, managing to shorten the reaction time from days to minutes. This considerable reduction in reaction time, in addition to speeding up the process, is a clear economic advantage.

One of the drawbacks in usage of CPs is their low aqueous solubility, which reduces fluorescence efficiency due to the formation of large aggregates that precipitate from the solution, severely limiting their biological applications. Accordingly, many efforts are being made to this end by developing strategies to improve the poor water solubility of these macromolecules [22]. A common strategy employed to increase solubility in aqueous media is based on the incorporation of ionic side chains into the main polymer chain obtaining conjugated polyelectrolytes (CPEs). Among CPEs, those containing fluorene units—named polyfluorenes—offer the advantage of excellent chemical and thermal stability, photostability, high fluorescence efficiency as well as a good synthetic accessibility with easy substitution at the fluorene C9 position. Bluish emission is common in fluorene-based CPEs, while copolymerization with other aromatic units change to longer wavelengths [23,24,25,26,27]. In this respect, our group has synthesized blue- and red-emitter cationic polyfluorenes, copoly-{(9,9-bis(6′-*N*,*N*,*N*-trimethylammonium)hexyl]-2,7-(fluorene)-*alt*-1,4-(phenylene)} bromide (HTMA-PFP) and copoly-{[9,9-bis(6′-*N*,*N*,*N*-trimethylammonium)hexyl]-2,7-(fluorene)-*alt*-1,4-(naphtho[2,3c]-1,2,5-thiadiazole)} bromide (HTMA-PFNT), which incorporate a phenyl and a naphtha[2,3c][1,2,5]thiadiazole group on fluorene backbone, respectively [28,29,30,31]. Both blue and red polyfluorenes have been characterized in aqueous media along with in the presence of biological systems. They show poor solubility in aqueous media, forming aggregates with low fluorescence efficiency but rapid incorporation into lipid membranes increasing their fluorescence signal and acting as fluorescent markers that allow the visualization of membrane structures, especially those mostly constituted by anionic lipids [12,30,32].

The use of green fluorescent probes in bioimaging seems to be less interesting than that of red probes due to their short penetration distance within tissue [33,34]. Though, when the surface is being imaged, green probes have considerable advantages over red ones due to their higher fluorescence emission efficiency and the ability to differentiate them from the background auto-fluorescence. Therefore, in order to complete the series of colour-tunable polyfluorenes, here in this work we describe the synthesis and further characterization of a new green-light emitting cationic CPE (HTMA-PFBT), which incorporates the chromophore 2-phenylbenzotriazole into the backbone of polyfluorene. As for HTMA-PFP and HTMA-PFNT, we have explored the ability of this new polyelectrolyte to be used as fluorescent membrane probe, comparing the results with those obtained with blue and red polyfluorenes. With this end, the behaviour of HTMA-PFBT was studied in aqueous media as well as in presence of model lipid membranes and bacterial and mammalian cells. An exhaustive study was realized with anionic and zwitterionic lipid vesicles and in samples that simultaneously contain both types of vesicles at different ratios, in order to explore the potential selectivity of HTMA-PFBT towards anionic systems. The study further investigates the potential use of the polyelectrolyte to detect changes in membrane phase properties.

## 2. Materials and Methods

### 2.1. Materials

Most of the chemical reagents and organic solvents used in this work were provided from Sigma-Aldrich Chemical Co (St. Louis, MO, USA) with the exception of the 9-bis(6-bromohexyl)-2,7-dibromofluorene compound which was supplied from SYNTHON GmbH & Co (Wolfen, Germany). Solvents of varying degrees of quality and purity have been used. In the synthesis reactions, for the purification of compounds and for the characterization of the molecular weight High Performance Liquid Chromatography (HPLC) grade solvents have been used. High-purity deuterated solvents were used for the Nuclear Magnetic Resonance (NMR) characterization and spectroscopic grade solvents (UVASOL, Merck, Darmstadt, Germany) have been used for the techniques of UV-visible and fluorescence spectroscopy. Sodium phosphate buffer 50 mM with 0.1 mM NaCl (pH 7.4) was prepared using distilled and deionized water with Milli-Q equipment (Millipore, Darmstadt, Germany). The fluorescent membrane marker 4,4-difluoro-5-(4-phenyl-1,3-butadienyl)-4-bora-3a,4a-diaza-s-indacene-3-undecanoic acid (BODIPY^®^ 581/591 C_11_) was obtained from Molecular Probes (Eugene, OR, USA). Stock solutions were prepared in ethanol (1 mM) and stored at −20 °C before use. The fluorescence quencher 9,10-Anthraquinone-2,6-disulfonic acid (AQS) was supplied from Sigma-Aldrich (St. Louis, MO, USA) and dissolved in water (5 mM), just before use.

The phospholipids l-α-Phosphatidylcholine (PC) and l-α-Phosphatidyl-dl-glycerol (PG) from egg yolk and synthetic phospholipids 1,2-Dimyristoyl-sn-glycero-3-phospho-rac-(1-glycerol) sodium salt (DMPG), 1,2-Dioleoyl-sn-glycero-3-phosphocholine (DOPC) 1,2-Dioleoyl-sn-glycero-3-phospho-rac-(1-glycerol) sodium salt (DOPG) and 1,2-Dipalmitoyl-sn-glycero-3-phospho-rac-(1-glycerol) sodium salt (DPPG) and used in this work were from Sigma-Aldrich (St. Louis, MO, USA) and all of them were used directly without further purification.

### 2.2. Synthesis and Characterization of Polymers

The monomer 2,7-(4,4,5,5-tetramethyl-[1,3,2] dioxaborolane)-9,9-bis(6′-bromohexyl)-fluorene (**M1**) was synthesized from compound 9,9-bis(6-bromohexyl)-2,7-dibromofluorene following the protocol previously described by Bazan et al. [26]. The monomer 4,7-dibromo-2-(phenyl)benzo [d] [1,2,3] triazole (**M2**) was synthesized and provided by the group of Professor María Pilar Prieto Núñez-Polo from the University of Castilla la Mancha [35].

#### 2.2.1. Synthesis of Copoly-{9,9-bis(6′-bromohexyl)-2,7-fluorene-*alt*-4,7-(2-(phenyl)benzo [d] [1,2,3] Triazole)} (**P1**)

Monomers, **M1** (0.3 mmol), **M2** (0.3 mmol) and tetrakis(triphenylphosphine)palladium(0) (0.005 mmol) were mixed in a microwave (MW) tube in an inert atmosphere. Subsequently, 4 mL of toluene was added together with 1 drop of Aliquat^®^ 336 (phase-transfer catalyst), the mixture was stirred until completely dissolved and 2 mL of potassium carbonate (water solution, 2 M) was added. Finally, it was left to react in a MW reactor under the following conditions: SPS method, 135 °C, T = 5 °C, 22 min and 150 W of power. The organic solvents were removed by evaporation and the product was solubilized in 2 mL of chloroform, precipitated in 250 mL of methanol and filtered under vacuum. The final precipitated product is left to dry under vacuum at 40 °C to give 120 mg yellow-green solid (yield: 59%). ^1^H NMR (500 MHz, CDCl_3_) (ppm): 8.61–7.20 (m, 13H, Ar); 3.25 (br, 4H, CH_2_–Br); 2.29–2.08 (br, 4H, CH_2_); 1.67 (br, 4H, CH_2_); 1.26–0.80 (br, 12H, CH_2_). ^13^C NMR (125 MHz, CDCl_3_) (ppm): 151.4 (C_Ar_), 144.7 (C_Ar_), 140.9 (C_Ar_), 140.5 (C_Ar_), 136.6 (C_Ar_), 130.8 (C_Ar_), 129.1 (CH_Ar_), 127.8 (CH_Ar_), 126.5 (CH_Ar_), 123.5 (CH_Ar_), 120.4 (CH_Ar_), 119.4 (CH_Ar_), 55.3 (C Fluoreno), 40.5 (CH_2_), 34.0 (CH_2_–Br), 32.8 (CH_2_), 29.8 (CH_2_), 27.9 (CH_2_), 24.9 (CH_2_). Fourier Transform Infrared (FTIR) (pellet BrK, cm^−1^): 3023, 2930, 2855, 1567 (s, C=N), 1468 (benzotriazole), 1387, 1266, 889, 821 (s, –(CH_2_)*_n_*–), 762, 641 (s, –CH_2_Br), 570, 529. GPC (PS calibration): *M_u_* = 683.5 g/mol; *M*w = 12,779 g/mol; *M_n_* = 4584 g/mol; *M_p_* = 8531 g/mol; *PDI* = 2.8.

#### 2.2.2. Synthesis of Copoly-{[9,9-bis(6′-*N*,*N*,*N*-trimethylammonium)hexyl]-2,7-(fluorene)-*alt*-4,7-(2-(phenyl)benzo[d][1,2,3]triazole)} bromide (HTMA-PFBT)

**P1** (0.11 mmol) was dissolved in tetrahydrofuran (50 mL) at −78 °C (dry ice-acetone bath) in an inert atmosphere. Meanwhile, in a different flask, a solution of trimethylamine in water was heated up to 50 °C. Trimethylamine (~7 mL) was added to **P1** in tetrahydrofuran drop by drop, by using a coldfinger condenser. The mixture was left stirring at room temperature for 24 h. Afterwards, 50 mL of water was added to the reaction flask and the previous procedure was repeated by adding more trimethylamine (~7 mL) and the mixture left stirring for 24 h at room temperature. The solvent and the excess of trimethylamine were evaporated. Approximately 80 mg of solid polymer with yellow colour was precipitated in acetone. Polymer was collected by filtration and dried under vacuum at 40 °C (yield: 95%). ^1^H NMR (500 MHz, CDCl_3_) (ppm): 8.61–7.20 (m, 13H, Ar); 3.27 (br, 4H, CH_2_–NR_4_); 3.08 (s, 18H, CH_3_); 2.19 (br, 4H, CH_2_); 1.65 (br, 4H, CH_2_) and 1.37–0.80 (br, 12H, CH_2_). FTIR (pellet BrK, cm^−1^): 3402 (s, NR_4_^+^), 3020, 2928, 2856, 1569 (s, C=N), 1483, 1466 (benzotriazole), 1400, 1285, 1264, 1235, 1060, 949, 820 (s, –(CH_2_)*_n_*–), 758.

#### 2.2.3. Microwaves

For the synthesis assisted in microwave, a CEM (Matthews, NC, USA) Discover microwave reactor with a magnetic stirring was used. The pressure sensor (IntelliVent) and the infrared (IR) sensor were helped monitoring and controlling the temperature. The reactions were carried out in standard Pyrex tubes of 10 mL. The method known as SPS (Solid Phase Synthesis) was performed, applying pulse of 150 W while maintaining the sample at a temperature of 135 ± 5 °C for 22 min.

#### 2.2.4. Nuclear Magnetic Resonance (NMR)

The NMR spectra were collected from a Bruker AVANCE 500 Hz (^1^H: 500 Hz; ^13^C: 125 Hz) spectrometer. Deuterated solvents containing tetramethylsilane (TMS) were used as an internal reference for the preparation of the samples. The chemical shifts (δ) were expressed in parts per million (ppm) and were adjusted to the reference TMS (δ = 0.00 ppm) or to the signal of the deuterated solvent itself. The spectra were analysed using the TopSpin 3.2 program (Bruker, Massachusetts, USA).

#### 2.2.5. Fourier Transform Infrared Spectroscopy (FTIR)

The FTIR spectra were recorded by using a Bruker IFS 66/S spectrometer (Billerica, MA, USA). The samples were analysed in the form of potassium bromide (KBr) pellets and the spectra were analysed between 400 and 4000 cm^−1^ at room temperature. The spectra were recorded with the OPUS program (Bruker) and analysed using the Spectra Manager (free software).

#### 2.2.6. Gel Permeation Chromatography (GPC)

The molecular weight distribution of the synthesized polymers was determined by using Gel Permeation Chromatography. Analysis was performed by using Shimadzu LC-20AD, with two detectors: Refractive Index Detector RID-10A (Shimadzu, Tokio, Japon) and Evaporated Light Scattering Detector (ELSD 3300, Alltech Associates, Büchi, Mexico). Two serially PLgel 5 µm MIXED-C columns and a guard column (PLgel 5 μm 50 × 7.5 mm) were used (Agilent, Santa Clara, CA, USA). Tetrahydrofuran (THF) was used as the mobile phase with the injection volume of 20 μL and 1 mL/min flow rate. Agilent polystyrene (EasiVials) standards were used for the calibration.

#### 2.2.7. Absorption and Fluorescence Measurements

Absorption measurements were carried out with a UV-1603 spectrophotometer (Shimadzu, Tokyo, Japan), at room temperature. A QuantaMaster spectrofluorometer (PTI, Birmingham, AL, USA) was used to record fluorescence spectra. Both, UV-visible and fluorescence spectra were performed in solution, using spectroscopic-grade solvents and 1 cm × 1 cm path length quartz cuvettes. The different samples of HTMA-PFBT polymer were excited at 425 nm. The Fluorescein dye (Fluorescein sodium salt obtained from Sigma-Aldrich (St. Louis, MO, USA) dissolved in NaOH 0.1 M) was used as a reference for fluorescence quantum yield measurements.

#### 2.2.8. Fluorescence Microscopy

The fluorescence microscopy images were recorded by using a Leica digital camera DFC3000G on a Leica DMI 3000B inverted microscope with Leica EL6000 compact light source (Wetzlar, Germany). Images were acquired using a 63× objective with filter FITR (Ex BP 480/40, Em BP 527/30) and DsRed (Ex BP 555/25, Em BP 620/60). Leica Application Suite AF600 Module Systems was used for the manual formatting and processing of data acquisition.

### 2.3. Studies of Interaction with Liposomes

#### 2.3.1. Large Unilamellar Vesicles (LUVs) Preparation

For the formation of liposomes, 3 mg of phospholipid was dissolved in chloroform and subsequently, the solvent was evaporated under argon (Ar), forming a thin lipid film at the bottom of the vial. The lipid film was then hydrated with 1 mL of phosphate buffer (50 mM, 0.1 mM NaCl, pH 7.4) at a temperature above the phase transition of the various phospholipids used and vortexed. Afterwards, the sample was passed through 0.1 μm polycarbonate filters to obtain large unilamellar vesicles (LUVs). The relevant amount of phosphate buffer was added until the desired lipid concentration reached.

#### 2.3.2. Giant Unilamellar Vesicles (GUVs) Preparation

Custom built Platinum electrode-containing Teflon chambers were used to prepared giant unilamellar vesicles (GUVs) by following an electroformation method [36,37]. They were composed of DOPG and DOPC labelled with BODIPY^®^ 581/591 C_11_ (probe: lipid ratio of 1:500). In brief, the suspension of phospholipids in chloroform was spread on the conductive surface of the electrodes. Afterwards, the organic solvent traces were removed under vacuum and lipid spread electrodes were then rehydrated with 450 μL of 200 mM sucrose solution in Milli-Q purified water. Finally, there was applied a cycle of 7 V voltage and 10 Hz frequency for 2 h followed by 7 V voltage and 1 Hz frequency for 30 min. Prepared GUVs were then collected and used before 48 h. To visualize the GUVs under the microscope, 200 μL of fresh 200 mM glucose solution were added into the microscopy well prior to 50 μL corresponding to the GUVs preparation.

#### 2.3.3. Preparation of HTMA-PFBT/Lipid Samples

Aliquots of the conjugated polyelectrolyte HTMA-PFBT, previously solubilized in dimethyl sulphoxide (DMSO, 6.2 × 10^−4^ M) and stored at −20 °C, were added to the liposome suspensions and the sample was incubated for 30 min at a temperature higher than the phospholipid phase transition. The final HTMA-PFBT concentration in the samples was 1.5 μM, in terms of repeat units, in most of the experiments. The proportion of DMSO was always kept lower than 1% (*v*/*v*) in the aqueous samples.

#### 2.3.4. Dynamic Light Scattering (DLS) Measurements

The size of the polyelectrolyte aggregates, as well as of the liposomes were measured by the Dynamic Light Scattering (DLS) technique. A Brookhaven 90 Plus particle size analyser (New York, NY, USA) equipped with LED/diode laser (35 mW) as a light source and with a 90° scattering angle of lecture was used for size measurements.

#### 2.3.5. Measurements of Partition Coefficient

To study the affinity of HTMA-PFBT for different lipid vesicles, the phospholipid/water partition coefficient (*K_P_*) was calculated. This parameter allows determining quantitatively the degree of lipophilicity of the polymer. The higher the *K_P_* value, the greater is the affinity of the polymer to the lipid vesicles.

Samples of LUVs with varying lipid concentration were prepared and a constant concentration of HTMA-PFBT was added to each sample, incubated for 30 min at 30 °C. Subsequently, the fluorescence spectrum was measured at 24 °C for each of the sample. To calculate *K_P_*, the following equation was used [38]:

(1) $$\Delta I=\frac{\Delta I_{max}\left[L\right]}{1/(K_{P}\gamma )}$$

where Δ*I* (Δ*I* = *I* − *I*_0_) corresponds to the difference between the fluorescence intensity (in other words, area of the emission spectrum) of the HTMA-PFBT measured in the presence (*I*) and absence (*I*_0_) of the lipid vesicles; Δ*I_max_* stands for the maximum value of this difference (Δ*I_max_* = *I_∞_ − I*_0_) once the limiting value is enhanced (*I_∞_*) by increasing the lipid concentration [*L*] and the phospholipid molar volume (*γ*) is 0.763 M^−1^ for fluid phases [39].

#### 2.3.6. Fluorescence Quenching Experiments

Fluorescence emission spectra in buffer of LUV-incorporated HTMA-PFBT were studied in the absence and presence of different concentrations of a fluorescence quencher. AQS is an electron acceptor acting as a fluorescence quencher for cationic polyelectrolytes through the formation of static quenching complexes, via electrostatic interactions [40,41]. Stern–Volmer analysis was used for the fluorescence quenching data according to the equation:

(2) $$\frac{I_{0}}{I}=1+K_{SV}\left[Q\right]$$

where *I*_0_ and *I* correspond to the steady-state fluorescence emission intensities in the absence (*I*_0_) and presence (*I*) of AQS; [Q] is the AQS concentration and *K_SV_* is the static Stern-Volmer constant [40]. The fluorescence quenching efficiency was calculated as *η* = (1 − *I/I*_0_) × 100.

In mixtures where anionic and zwiterionic LUVs coexist, the theoretical quenching efficiency (*η_expected_*) which assume that HTMA-PFBT shows similar affinity towards PC and PG LUVs was determined from the fluorescence intensities in the absence *I*_0_*^t^* and presence *I^t^* of the quencher by using the following equation:

*η_expected_* = (1 − *I^t^*/*I*_0_*^t^*) × 100 (3)

The values expected for these intensities can be estimated as:

*I*_0_^*t*^ = (*χ^PC^ Φ^PC^*) + (*χ^PG^ Φ^PG^*) (4)

*I^t^* = (*χ^PC^ Φ^PC^* (1 − *η^PC^*/100)) + (*χ^PG^ Φ^PG^* (1 − *η^PG^*/100)) (5)

*χ^PC^* and *χ^PG^* are the lipid molar fraction of the zwitterionic and anionic model systems in the mixture, respectively, while *Φ^PC^* and *Φ^PG^* correspond to the quantum yield of HTMA-PFBT either in PC or PG LUVs, respectively. The quenching efficiencies *η^PC^* and *η^PG^* represent the percentage of quenching, for a fixed concentration of quencher, when the sample contains 100% of PC or PG LUVs, respectively.

### 2.4. Bacterial and Mammalian Cell Imaging

For the experiments with bacteria and mammalian cells fluorescence microscopy images were recorded in absence and in presence of HTMA-PFBT (0.2 µM), separately with human HeLa cells and *Escherichia Coli* (*E. coli*) Top 10 F’ strain. The human cell line HeLa was cultured at 37 °C under a 5% CO_2_ atmosphere in high glucose (4.5 g/L) Dulbecco’s modified eagle’s medium (DMEM) containing Foetal Bovine Serum (10%), l-glutamine (2 mM) and antibiotics (100 U/mL penicillin and 0.1 mg/mL streptomycin; Sigma Aldrich). Shortly before the microscopy imaging, HeLa cells were trypsinized and subsequently resuspended in phosphate buffered saline (PBS) at final concentration of 10^5^ HeLa cells per mL. *E. coli* Top 10 F’ strain was grown overnight at 37 °C in LB medium. Then, 20% glycerol stock aliquots were prepared, stored at −80°C and tittered, as number of colony forming units (CFUs) per mL, prior to use. The final concentration of bacteria in the experiments with HTMA-PFBT was 10^6^ CFUs/mL.

## 3. Results and Discussion

### 3.1. Synthesis and Characterization of HTMA-PFBT

**Scheme 1:**
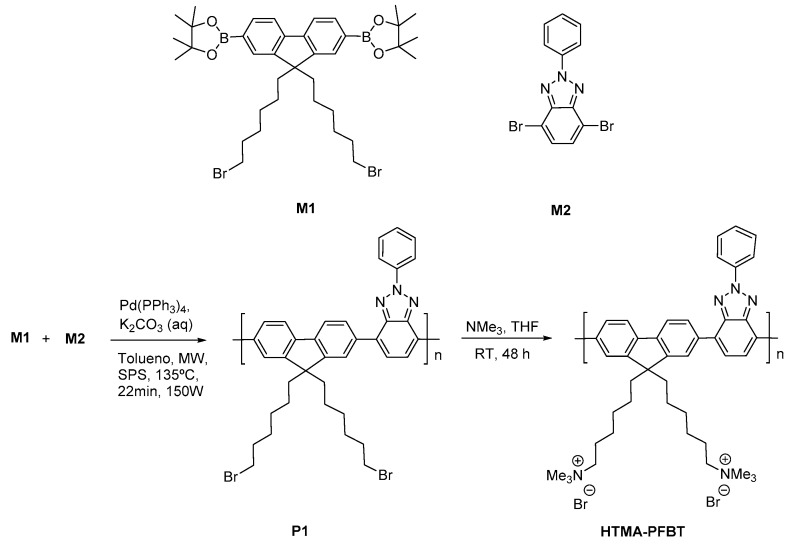
Synthetic Approach of Copoly-{[9,9-bis(6′-*N*,*N*,*N*-trimethylammonium)hexyl]-2,7-(fluorene)-*alt*-4,7-(2-(phenyl)benzo[d][1,2,3]triazole)} bromide (HTMA-PFBT).

The polyelectrolyte HTMA-PFBT was synthesized from the neutral polymer precursor **P1** (Scheme 1) by a quaternization reaction (Menschutkin Reaction). **P1** was synthesized from the monomers **M1** and **M2** by a Suzuki cross-coupling reaction carried out in a microwave reactor. Microwave-assisted synthesis allowed us to significantly reduce the reaction time compared to other analogous polymers previously synthesized by our research group from 48 h to only 22 min [28,30,42]. For the synthesis of **P1**, the method known as Solid Phase Synthesis (SPS) was used, because it provides the best results in the synthesis of CPs in microwaves, as indicated in previous references [19,43] As a final product, a solid with yellow-green colour was obtained with a yield of 59%. Analysis by gel permeation chromatography (GPC) of **P1** showed a weight-average molecular weight (*M*w) of 12,779 g/mol with a polydispersity index (*PDI* = *M*w/*M_n_*) of 2.8. The subsequent post-polymerization reaction on the polymer **P1** allowed us to obtain the polyelectrolyte of interest with 95% yield. Similar to other analogous polyfluorenes, the results obtained by NMR and FTIR spectroscopies are consistent with the structure of **P1** and demonstrate that the subsequent modification to obtain the polyelectrolyte HTMA-PFBT has been successfully carried out.

**Figure 1:**
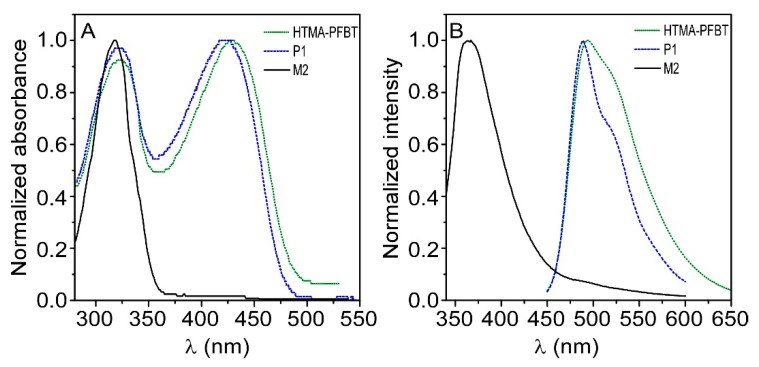
Normalized absorption (**A**) and fluorescence emission spectra (**B**) of **M2** (black) and **P1** (blue) in Tetrahydrofuran (THF) and HTMA-PFBT (green) in ethanol. Excitation wavelength (λ_exc_) was 320 nm for **M2** and 425 nm for **P1** and HTMA-PFBT.

UV-Vis normalized absorption spectra of monomer 2-phenylbenzotriazole, **M2** and polymer **P1** in THF showed clear differences (Figure 1A). The absorption spectrum of **M2**, recorded between 290 and 550 nm, displayed one characteristic absorption peak at 317 nm, while the spectrum of **P1** exhibited two absorption bands at 322 and 424 nm, which were different than those corresponding to chromophores fluorene and 2-phenylbenzotriazole, separately. The fluorescence spectra of **M2** and **P1** also displayed a different emission pattern, as is shown in Figure 1B. **M2** presented the emission maximum at 367 nm, while **P1** exhibited its maximum peak around 490 nm, showing a large Stokes shift. In addition, excitation spectrum of **P1** was similar to the absorption one and neither the position nor the shape of the emission spectrum was affected by the excitation at different wavelength (data not shown). All these results indicate that an effective conjugation is taking place between fluorene and 2-phenylbenzotriazole in P1, leading to the green emission band observed at 500 nm.

The further derivation of **P1** to obtain the polyelectrolyte HTMA-PFBT produced a slight red-shift in the absorption and emission maxima besides the widening of the emission spectrum. This pattern might be attributed to differences in the conformation of the polymer chains because the incorporation of the cationic groups, as well as to the different solvent employed (HTMA-PFBT was dissolved in ethanol where it is more soluble).

### 3.2. HTMA-PFBT in Aqueous Solvents

**Figure 2:**
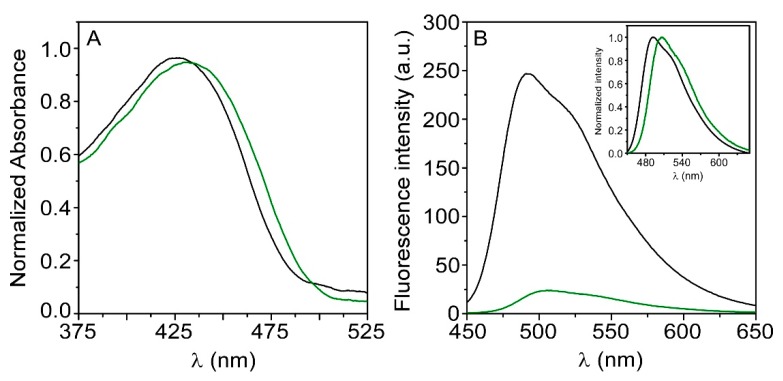
(**A**) Normalized Absorption spectra of HTMA-PFBT (3.1 μM in terms of repeat unit) in ethanol (black) and buffer (green). (**B**) Fluorescence emission spectra of HTMA-PFBT (1.5 μM) in ethanol (black) and buffer (green). Inset: Normalized fluorescence emission spectra of HTMA-PFBT in ethanol (black) and buffer (green). (λ_exc_ = 425 nm).

In order to check the potential use of HTMA-PFBT as a green-emitting membrane probe, its behaviour in aqueous media was studied. The absorption and emission properties of the polyelectrolyte were explored in a phosphate buffer (50 mM with NaCl 100 mM at pH 7.3) to mimic physiological conditions and compared to those obtained in ethanol, at the same conditions. Results are shown in Figure 2 and Table 1. Absorption spectra were recorded between 280 and 530 nm, although Figure 2 only shows the spectra recorded in the visible region. In ethanol, a clear absorption band with a maximum at 425 nm was observed. The absorbance at this wavelength was linearly increasing with polymer concentration and, following the Lambert-Beer law, a molar absorption coefficient of ε^425^ = 14,500 M^−1^ cm^−1^ was calculated. When HTMA-PFBT was added to the buffer, the absorption spectrum was shifted to the red by about 6 nm (Figure 2A) and a very small decrease in the absorbance value was detected. Regarding the fluorescence properties, the most remarkable result was the strong decrease in the fluorescence intensity of the polyelectrolyte observed in buffer (Figure 2B). The fluorescence quantum yield was reduced from 0.22, measured in ethanol, to 0.02 upon incorporation in the aqueous medium. In addition, the spectrum was clearly shifted to the red (~17 nm) respect to that recorded in ethanol (See inset in Figure 2B and Table 1). This behaviour evidences the low solubility of HTMA-PFBT in buffer respect to that observed in ethanol and suggests the possible existence of aggregates formed by self-assembly of the polymer aromatic backbones, as has been reported for HTMA-PFP and HTMA-PFNT [12,30,44].

The formation of such aggregates was confirmed by DLS experiments. The results in Table 1 show that polymer aggregates of around 200 nm hydrodynamic diameters were formed in buffer, while no aggregation was detected in ethanol. Furthermore, the average size of these aggregates increased over time and reached sizes of around 340 nm in one hour with a corresponding decrease in their fluorescence intensity (data not shown). This behaviour, common to the other CPEs, has been attributed to nonspecific electrostatic interactions between the quaternary ammonium groups and the anionic species of the buffer solution leading to neutral complexes which aggregate in different supramolecular structures [45].

### 3.3. HTMA-PFBT in Model Membranes

**Figure 3:**
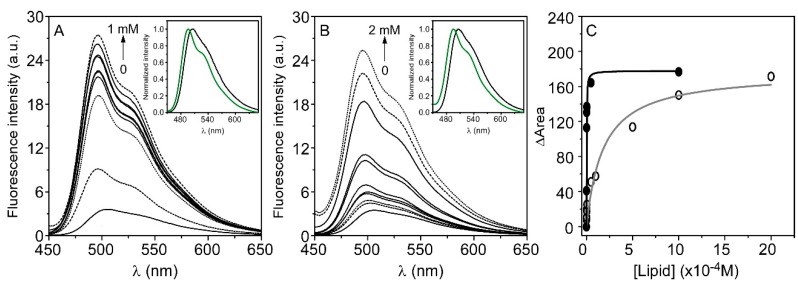
Fluorescence emission spectra of HTMA-PFBT (1.5 μM) in buffer and at increasing concentrations of LUVs of PG (**A**) and PC (**B**). Insets in both (**A**,**B**) represent the normalized fluorescence emission spectra of HTMA-PFBT in buffer (black) and incorporated in LUVs (green). (All recorded upon λ_exc_ = 425 nm). (**C**) Area of the fluorescence emission spectrum of HTMA-PFBT at different concentrations of LUVs of PG (black circles) and PC (hollow circles).

Once HTMA-PFBT was characterized in buffer, its potential use as fluorescent membrane marker was explored using model membranes of anionic and zwitterionic lipids. With this end, samples of LUVs with increasing final lipid concentrations from 0 to either 1 mM (for PG) or to 2 mM (for PC) were prepared in buffer. Then, HTMA-PFBT up to a final concentration of 1.5 µM (in terms of repeat units) was added to each one at room temperature. The interaction of the polyelectrolyte with the lipid vesicles was explored by the changes occurring in its fluorescence emission spectra (Figure 3, Table 1). A sharp increase in the fluorescence intensity, together with a blue-shift and a narrowing of the emission spectrum were observed in the presence of increasing concentrations of both, anionic and zwitterionic vesicles, as compared to buffer, although the fluorescence intensity was much more sensitive to the presence of low concentrations of PG. In addition, a higher resolution in the vibrational structure was detected, which could be an indicator for the polymer chains having a decrease in the number of degrees of freedom because of the interaction with the lipid membranes and hence a reduction in the number of conformations existing in the excited state. All these results suggest the breaking of aggregates and the incorporation of the polyelectrolyte into the lipid bilayer. Figure 3C show plotting the area under each of the emission spectrum of the HTMA-PFBT obtained for the different lipid concentrations. The partition coefficient between the lipid and the aqueous phase (*K_P_*) of the conjugated polyelectrolyte was calculated using Equation (1), from a two-parameter (Δ*I_max_* and *K_P_*) fitting procedure, which values are summarized in Table 2. From this fit, a value of *K_P_* = 9.4 × 10^5^ was obtained for the anionic vesicles, which indicates a very high affinity of HTMA-PFBT for this type of membranes, presumably because of the electrostatic interactions between the ammonium groups of the polyelectrolyte and the PG polar heads. This value was very similar to that obtained for the red polyelectrolyte HTMA-PFNT and higher than that calculated for the blue one [12,30,44,46]. In the case of the PC LUVs, the *K_P_* value was ~100 times lower than that corresponding to the PG vesicles, evidencing the higher preference of HTMA-PFBT to anionic membranes. The fluorescence quantum yields were estimated for these samples obtaining high values, close to 0.5, which were 25 times larger than that obtained in buffer and twice higher than in ethanol (Table 1).

**Figure 4:**
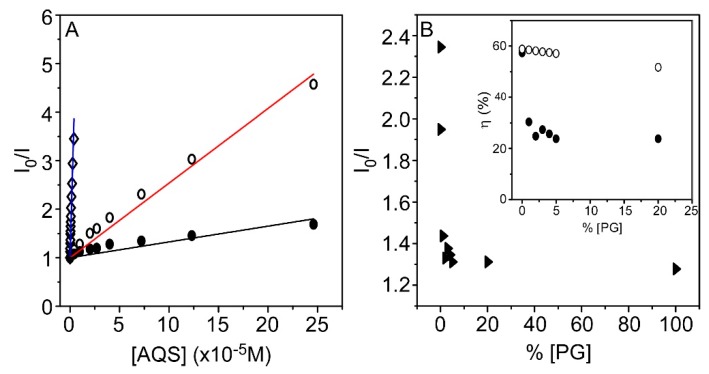
(**A**) Stern-Volmer plots for quenching of HTMA-PFBT (1.5 μM) by AQS in buffer (hollow diamonds) and in LUVs of PG (black circles) and PC 1 mM (hollow circles). (**B**) Quenching induced by AQS (100 μM) in the fluorescence intensity of HTMA-PFBT in samples containing PC LUVs with increasing concentrations of PG LUVs. Inset: Experimental quenching efficiency *versus* increased PG vesicles percentage (black circles) compared with the theoretical quenching efficiency determined if HTMA-PFBT had the same preference for both types of vesicles (hollow circles).

In order to explore if the insertion of the polyelectrolyte into LUVs could alter their size and/or stability, the hydrodynamic diameters of the vesicles were estimated by DLS measurements, before and after addition of HTMA-PFBT. In absence of polyelectrolyte, a single distribution centred at ~158 nm and ~140 nm was observed for the LUVs of PC and PG, respectively (Table 1). When HTMA-PFBT 1.5 µM was added to these lipid suspensions, similar single distributions were obtained and no additional population was detected, although the hydrodynamic diameter of PC LUVs was slightly larger than that observed in absence of the polyelectrolyte (Table 1). These results confirm that no polymer aggregates were found in the buffer and thus all the polymer chains might be interacting with lipid vesicles, which integrity is maintained even after HTMA-PFBT incorporation. In addition, the fact that only the size of the PC LUVs and not that of PG, was slightly increased in presence of polyelectrolyte suggests a different location of the polymer chains in the zwitterionic and anionic membranes. This hypothesis was also checked with the quenching experiments using the electron acceptor AQS, which is a water-soluble quencher remaining out of the lipid bilayer. The quenching efficiency was explored for HTMA-PFBT in both PC and PG LUVs and compared to that obtained for the CPE in buffer. Results showed that the higher the concentrations of the quencher were, the lower fluorescence intensity was observed and this decrease was much more pronounced in buffer than in the lipid suspensions. In the anionic system, only a minor decrease in the fluorescence emission signals of the polyelectrolyte was observed, while the quenching effect was higher in the zwitterionic model. Figure 4A shows the Stern-Volmer plots of these experiments. Plots were lineal in the concentration range explored but the quenching efficiencies were clearly different in the three systems. Table 2 shows the *K_SV_* values determined from the slope of the plots, following Equation (2). The fact that the quenching effect and thus the *K_SV_* value is lower for HTMA-PFBT in the lipid suspensions than in the buffer indicates its protection from AQS and hence its incorporation into the lipid bilayer. However, the *K_SV_* value determined in PC was about 6 times higher than that obtained in PG, suggesting that HTMA-PFBT remains located on the membrane surface in the zwitterionic system, while it penetrates more deeply into the lipid bilayer of the anionic vesicle. This final location probably explains why the diameter of the PC LUVs increases slightly after the incorporation of the polyelectrolyte, while it remains unaltered in the PG LUVs.

### 3.4. Selectivity of HTMA-PFBT against Anionic and Zwitterionic Model Membranes

The results obtained for PC and PG LUVs suggest a different interaction mechanism between the polyelectrolyte and the lipid membrane which is dependent on the lipid charge of the vesicles. HTMA-PFBT shows higher affinity to the anionic membrane which is mainly attributed to electrostatic interactions, however, the final location into the lipid bilayer indicates that hydrophobic forces contribute to its solubilisation as well. In contrast, the affinity of the polyelectrolyte to zwitterionic membrane is clearly lower than that observed in the anionic system because the variation in the membrane surface net charge. The fact that HTMA-PFBT locates preferably on the membrane surface of PC vesicles also evidences a lower solubilisation and probably a lower breaking of aggregates. This different behaviour suggests the hypothesis of the polyelectrolyte being selective for anionic over zwitterionic membranes as has been reported for HTMA-PFP and HTMA-PFNT [44,46]. The differences observed in the quenching efficiency of the polymer in PG and PC vesicles were used to confirm this hypothesis. With this propose, lipid samples were prepared containing PC LUVs and increasing concentrations of PG LUVs (the final total lipid concentration was always 1 mM, increasing the PG LUVs ratio from 0% to 100%). The concentration of HTMA-PFBT (1.5 µM) and AQS (100 µM) was kept constant and added to each sample and the fluorescence spectra were recorded in order to determine the quenching efficiency (*η*) for the different samples. This parameter was calculated from the fluorescence intensities measured in absence (*I*_0_) and presence (*I*) of the quencher, as is described in Methods. Figure 4B and its inset illustrate, respectively, the experimental decrease observed in *I*_0_*/I* and *η*, as a function of increased PG vesicle content. According to these plots, the quenching efficiency for the sample containing either only PC LUVs or only PG LUVs is approximately 60 and 20%, respectively. The inset plot (Figure 4B) helps to confirm the theoretical expected quenching efficiency if HTMA-PFPBT had the same preference for PG and PC membranes. This value was estimated from Equations (3)–(5), as is described in Methods. Results show that the quenching efficiency determined experimentally is much lower than that expected even at very low concentrations of the anionic vesicles. It indicates that at these conditions the polyelectrolyte is preferentially embedded in the anionic membrane and thus protected from the quencher. In fact, a very slight increase in the proportion of PG LUVs rapidly decreases the quenching efficiency, showing similar efficiencies a sample containing only PG LUVs than a sample with 95% of zwitterionic lipids and 5% of anionic ones. This study confirms, as was previously suggested from the partition coefficients, that HTMA-PFBT shows selectivity towards anionic membranes over zwitterionic ones.

**Figure 5:**
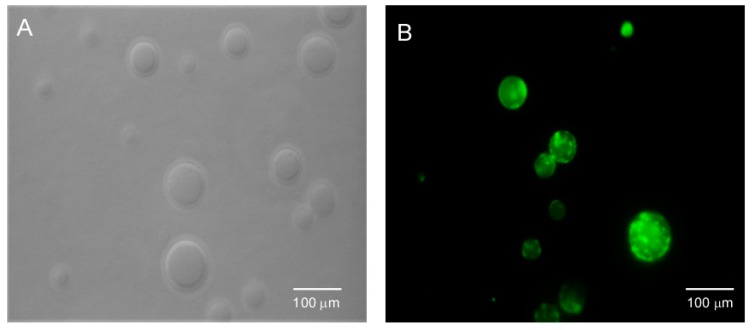
(**A**). Phase contrast image of 1,2-Dioleoyl-sn-glycero-3-phosphocholine (DOPC), giant unilamellar vesicles (GUVs) in absence of HTMA-PFBT. (**B**) Fluorescence microscopy image observed after addition of the polyelectrolyte to the previous sample in different field, recorded upon excitation with Vis-light using the FITR filter set.

The above results suggest the potential use of HTMA-PFBT to specifically target anionic lipid head groups. To check this hypothesis, we firstly explored under phase contrast and fluorescence microscopy, whether HTMA-PFBT was able to label giant liposomes (GUVs). Figure 5 shows the image of GUVs of the phospholipid DOPG recorded before and after addition of the polyelectrolyte. In absence of HTMA-PFBT (Figure 5A), vesicles were observed by phase contrast microscopy and no fluorescence was detected once the samples were excited with Vis-light (data not shown). However, when HTMA-PFBT was added to the same sample, GUVs fluorescing in green were clearly visualized upon visible light illumination (Figure 5B). Similar results were obtained when GUVs of DOPC, instead of DOPG, were prepared (data not shown). The fact that the shape and size of these GUVs are preserved after polymer addition, confirms that HTMA-PFBT at low concentrations spontaneously associates to the lipid vesicles without altering their spherical structure.

**Figure 6:**
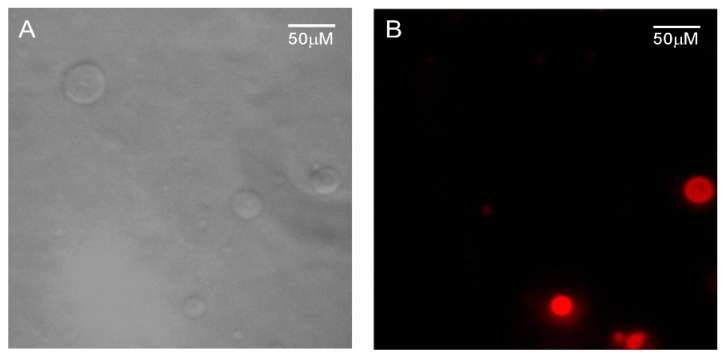
Microscopy images of DOPG GUVs in a mixture with DOPC-BODIPY GUVs obtained with (**A**) phase contrast and (**B**) upon excitation with Vis-light using the Leica DsRed filter (both images correspond to the same field).

**Figure 7:**
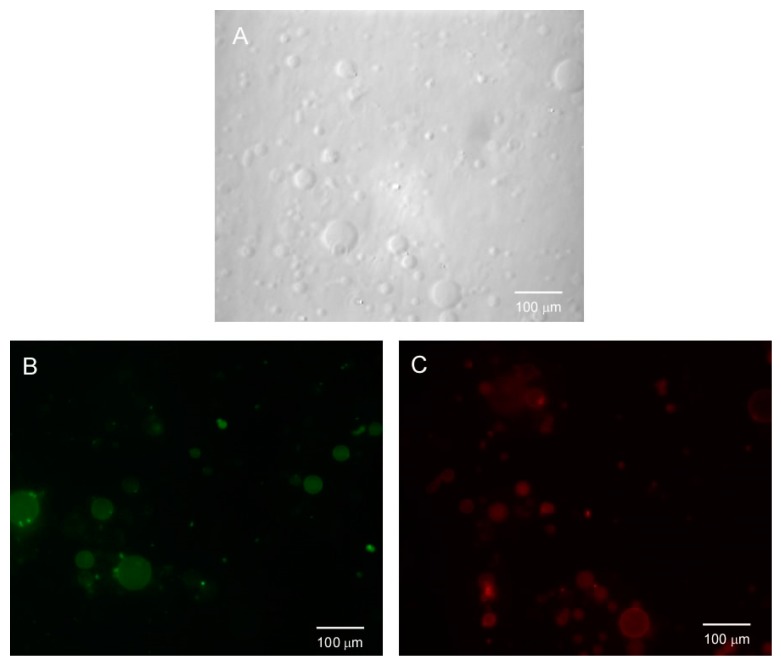
Fluorescence microscopy images of a mixture of DOPG and DOPC-BODIPY GUVs obtained after HTMA-PFBT addition, observed by phase contrast (**A**) and upon excitation with Vis-light using the FITR filter set (**B**) or the DsRed filter set (**C**). All images correspond to the same field.

Once it was proven the ability of the polyelectrolyte to image membrane structures, a comparative study was carried out for two series of GUVs prepared at the same lipid concentration but containing anionic or zwitterionic phospholipids. Anionic GUVs were composed of DOPG, while the zwitterionic vesicles were made with DOPC and labelled with the red-fluorescent probe BODIPY 581/591 C_11_. Equivalent volumes of both samples were placed on a well slide and microscopy images were taken before (Figure 6) and after HTMA-PFBT addition (Figure 7). As expected, in absence of polyelectrolyte both types of GUVs were observed in phase contrast (Figure 6A) but only the fluorescence of some of them (the labelled-DOPC GUVs) was visualized under excitation with the DsRed filter set (Figure 6B).

Figure 7 shows fluorescence microscopy images corresponding to the same field and obtained after addition of HTMA-PFBT and upon excitation using either the FITR filter set (Figure 7B) or the DsRed filter set (Figure 7C) (Figure 7A corresponds to the image observed by phase contrast). Images show that the GUVs that fluoresce in green are different from those fluorescing in red. This result is direct evidence that HTMA-PFBT selectively labels the anionic vesicles. The fact that the bacterial membrane is rich in phospholipids with anionic head group, makes HTMA-PFBT a suitable candidate to detect bacterial cells by targeting selectively the bacterial membrane over the mammalian cells, which have mostly zwitterionic membrane surface, as was demonstrated for the red polyelectrolyte HTMA-PFNT [46].

### 3.5. HTMA-PFBT as A Cell Membrane Marker

**Figure 8:**
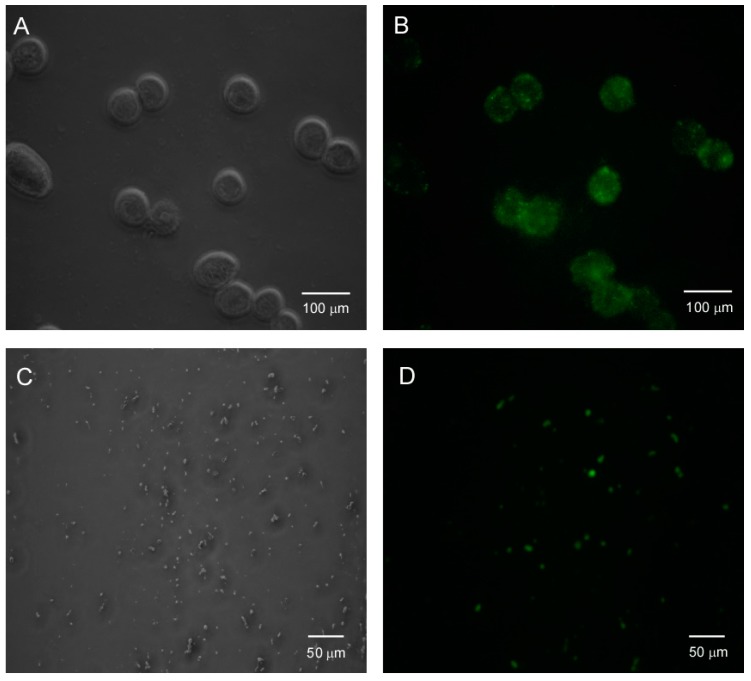
Microscopy images of human HeLa cells (**A**,**B**) and bacteria *E. coli* (**C**,**D**) in presence of HTMA-PFBT, observed by contrast phase (**A**,**C**) and upon excitation with Vis-light using the FITR filter set (**B**,**D**).

Preliminary qualitative experiments were performed to explore the ability of HTMA-PFBT to label alive bacterial (*E. coli* Top 10 F’ strain) and mammalian cells (human HeLa cells). The microscopy images were taken 5 min after the addition of HTMA-PFBT at a final concentration of 0.2 µM. Figure 8A,B correspond to the same field and show, respectively, the phase contrast image and the fluorescence image of the cells upon excitation with Vis-light, using the FITR filter cube. Results show that the polyelectrolyte rapidly interacts with the cells, permitting their visualization under fluorescence microscopy. Similar results were obtained with bacteria *E. coli* and are shown in Figure 8C,D. The images show the capability of this green polyelectrolyte to label both bacterial and mammalian cells. In addition, HTMA-PFBT was photostable, that is, preserved its fluorescence during the acquisition of the images without experiencing photobleaching.

### 3.6. HTMA-PFBT as a Fluorescent Probe to Detect Lipid Phase Transitions

**Figure 9:**
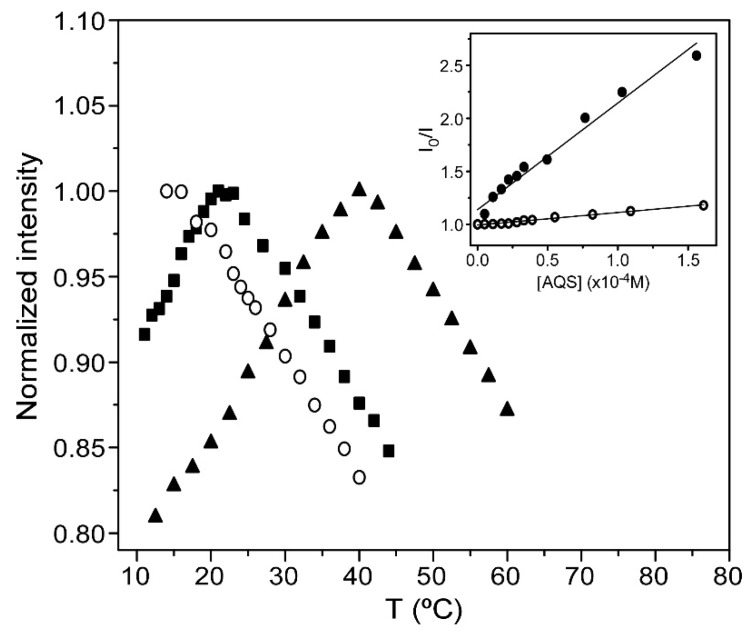
Effect of temperature on the fluorescence emission maximum of HTMA-PFBT in LUVs of DOPG (circles), 1,2-Dimyristoyl-sn-glycero-3-phospho-rac-(1-glycerol) sodium salt (DMPG) (squares) and 1,2-Dipalmitoyl-sn-glycero-3-phospho-rac-(1-glycerol) sodium salt (DPPG) (triangles). Inset: Stern-Volmer of the quenching of HTMA-PFBT with AQS in DMPG LUVs at 15 °C (circles) and 35 °C (hollow circles).

The above results indicate the potential use of the polyelectrolyte as a fluorescent marker to label cells that can selectively target anionic membranes. But, fluorescent probes can also be used to monitor the physical state of the membrane always that their fluorescent properties are sensitive to the different lipid phases. In cells, lipids can adopt various phases, which are characterized by a different spatial arrangement and motional freedom of each lipid with respect to its neighbour. The study of the phase behaviour of lipid bilayers is of essential importance in membrane research because it is involved in a great variety of membrane processes [47]. The more frequently perturbing variable used to study lipid-phase transitions is temperature [48]. The effect of temperature on lipid phase transitions is currently studied by fluorescence probe techniques, using fluorophores having photophysical properties sensitives to the fluidity and packing order [37]. It is known that the fluorescence emission of certain conjugated polyelectrolytes may be altered by any process involving conformational changes of the polymer chains or aggregation [12,44]. Therefore, this property could be used as a tool to monitor alterations on the physical state of the lipid membranes. In this sense, we reported in previous works the ability of the blue HTMA-PFP to detect lipid phase changes [12,44] and more recently, the Evans group reported the use of an anionic polythiophene to sequentially detect the phase transitions occurring within model phospholipid bilayers [9]. Taking into account these results, we decided to explore if the fluorescent properties of HTMA-PFBT were sensitive to the physical state of lipid bilayers. With this end, LUVs of three different phospholipids, DOPG, DMPG and DPPG, were prepared. These lipids present their gel/fluid phase thermal transition (T_m_) around −18 °C, 23 °C and 40 °C, respectively [49]. Below this temperature, lipids exist as a solid-like gel with their acyl chains tightly packed against each other. On the contrary, above T_m_, lipids are in a fluid phase state where the acyl chains are disordered and rapid lateral diffusion of the molecules occurs in the plane of the membrane. With this in mind, an aliquot of HTMA-PFBT was added to the three vesicle suspensions (1.5 μM final concentration) and the fluorescence emission spectra were recorded as a function of temperature. Significant changes in the fluorescence intensity were detected with increasing temperature but the spectral position remained unaltered. Figure 9 shows the dependence with the temperature of the intensity maximum for the three samples. For DOPG LUVs, the fluorescence intensity is more or less monotonically decreasing in the explored range of temperatures. This behaviour is expected because DOPG is an unsaturated lipid that does not show phase transition above 0 °C. Therefore, intensity decreases as the temperature rises since the probability of nonradiative transitions also increase with temperature. In contrast, for DMPG LUVs, it is possible to see an approximately linear increase in intensity between 10 °C and 22 °C, followed by a stabilization between 22–23 °C and a subsequent decrease at higher temperatures, which is practically parallel to that observed for DOPG. Finally, fluorescence intensities measured in DPPG LUVs follow the same pattern as in DMPG but with its maximum value near 40–41 °C.

The gel/fluid phase thermal transitions occurring within the DMPG and DPPG lipid bilayer may be responsible of this behaviour. Probably, the continuous increase in fluorescence intensity with increasing temperature in the gel phase is consequence of a better “solubilization” of the polyelectrolyte in the bilayer. It could be due to the effect of temperature on the fluidity and packing order of the membrane, which are respectively related to the rate and extent of phospholipid acyl chain motions away from an average orientation. As consequence of these dynamical and structural alterations, the polyelectrolyte chains can penetrate easier into the bilayer leading an increase in the fluorescence intensity. Once reached the transition temperature, the polymer attains a stable location within the bilayer and therefore the fluorescence signal is stabilized. The subsequent decrease in intensity above this temperature could be explained, as for DOPG, because the non-radiative pathways become more probable at higher temperatures, as has been reported for other conjugated polyelectrolytes [30,45,50]. To support this assumption, an additional fluorescence quenching experiment was performed with AQS, for HTMA-PFBT in LUVs of DMPG at 15 and 35 °C. Fluorescence intensity was recorded at increasing quencher concentrations (up to 1.6 × 10^−4^ M) and the Stern-Volmer plots are shown in Figure 9 (inset). The *K_SV_* value determined from the slope of these plots in fluid phase was around 8.5 times lower than in gel phase, evidencing that the polyelectrolyte is more protected from the quencher in the fluid phase than in the gel phase and, therefore, is more embedded in the lipid bilayer.

These results show that HTMA-PFBT could be used not only as a fluorescent imaging probe able to selectively label anionic membrane but also as a tool to monitor lipid-phase transitions.

## 4. Conclusions

In this study, the new green-emitter cationic conjugated polymer HTMA-PFBT, has been synthesized by microwave-assisted Suzuki coupling reaction, in order to complete the series of colour-tunable polyfluorenes synthesized in our laboratory. The polyelectrolyte has been characterized in aqueous media, where it shows poor solubility, forming aggregates with low fluorescence efficiency. Interaction of HTMA-PFBT with anionic and zwitterionic lipid vesicles induces the breaking of polymer aggregates, eliminating interchain quenching and increasing its fluorescence efficiency. This polyelectrolyte shows a strong preferential specificity for anionic membranes over zwitterionic membranes, probably due to electrostatic interactions, as well as a more internal location within the bilayer. In this sense and based on our previous studies, HTMA-PFBT shows a higher preference for anionic membranes than the blue polyelectrolyte (HTMA-PFP) and very similar to that determined for the red polyelectrolyte (HTMA-PFNT), what makes it a suitable candidate for potential bioimaging applications, as it has been reported for HTMA-PFNT. Fluorescence microscopy studies evidence the interaction of HTMA-PFBT with the lipid vesicles, labelling the lipid bilayer without perturbing its morphology and confirm the clear preference of the polyelectrolyte towards anionic systems. This behaviour suggests the potential use of HTMA-PFBT as a green cellular marker for the detection of different malignancies. On one hand, the fact that the bacterial membrane is rich in anionic lipids makes HTMA-PFBT a suitable candidate for bioimaging and selective recognition of bacteria over mammalian cells, as has been reported for the red polyfluorene HTMA-PFNT. On the other hand, it is known that the healthy mammalian cell plasma membrane has an asymmetric distribution of phospholipids and that during cell death, cell activation, or cell transformation the outer membrane surface becomes anionic due to the exposure of negative lipids. Therefore, HTMA-PFBT could be used to selectively target the surfaces of proliferating mammalian cancer cells. Finally, results show that the fluorescence emission properties of HTMA-PFBT depend on the physical state of the lipid bilayer. It suggests that the polyelectrolyte could function as an interesting tool to monitor changes in physical state of membranes.

## Figures and Tables

**Table 1:** Absorption, fluorescence characterization and hydrodynamic diameter of HTMA-PFBT in ethanol, buffer, Large Unilamelar Vesicles (LUVs) of phosphatidylglycerol (PG) and LUVs of phosphatidylcholine (PC).

-	Absorbance	Emission			d (nm) ^a^	
	λ_max_ (nm)	λ_max_ (nm) Φ_F_ BW (nm)			HTMA-PFBT + HTMA-PFBT	
**ethanol**	425	491	0.22	78	not detected	not detected
**buffer**	431	508	0.02	76	not detected	207 ± 4
**PC 1 mM**	-	496	0.45	65	158 ± 1	163 ± 1
**PG 1 mM**	-	495	0.47	64	140 ± 1	140 ± 1

^a^ The polydispersity index of the samples was <0.15.

**Table 2:** Partition coefficient, *K_P_*, values obtained from the fit of Equation (1) and Stern Volmer constants, *K_SV_*, determined from Equation (2) for HTMA-PFBT in buffer and in anionic and zwitterionic lipid vesicles in presence of 9,10-Anthraquinone-2,6-disulfonic acid (AQS)

System	*K_P_*	*K_SV_* (M^−1^)
**buffer**	-	(7.2 ± 0.4) × 10^5^
**PG**	(9.4 ± 2.2) × 10^5^	(2.6 ± 0.3) × 10^3^
**PC**	(8.9 ± 3.2) × 10^3^	(1.4 ± 0.1) × 10^4^
